# The cutting edge of archaeal transcription

**DOI:** 10.1042/ETLS20180014

**Published:** 2018-11-14

**Authors:** Thomas Fouqueau, Fabian Blombach, Gwenny Cackett, Alice E. Carty, Dorota M. Matelska, Sapir Ofer, Simona Pilotto, Duy Khanh Phung, Finn Werner

**Affiliations:** RNAP laboratory, Institute of Structural and Molecular Biology, Division of Biosciences, University College London, Gower Street, London WC1E 6BT, U.K.

**Keywords:** archaea, Elf1, evolution, NusA, RNA polymerase, transcription

## Abstract

The archaeal RNA polymerase (RNAP) is a double-psi β-barrel enzyme closely related to eukaryotic RNAPII in terms of subunit composition and architecture, promoter elements and basal transcription factors required for the initiation and elongation phase of transcription. Understanding archaeal transcription is, therefore, key to delineate the universally conserved fundamental mechanisms of transcription as well as the evolution of the archaeo-eukaryotic transcription machineries. The dynamic interplay between RNAP subunits, transcription factors and nucleic acids dictates the activity of RNAP and ultimately gene expression. This review focusses on recent progress in our understanding of (i) the structure, function and molecular mechanisms of known and less characterized factors including Elf1 (Elongation factor 1), NusA (N-utilization substance A), TFS4, RIP and Eta, and (ii) their evolution and phylogenetic distribution across the expanding tree of Archaea.

## Introduction

Transcription — the DNA template-dependent synthesis of RNA — is essential to life. The overall structure of the molecular machine that drives transcription, RNA polymerase (RNAP), is universally conserved in all domains of life, including Bacteria, Archaea and Eukarya. But whereas bacteria and archaea use a single RNAP to transcribe all genes, eukaryotes have compartmentalized the transcription space into distinct subsets of genes that are transcribed by three and five different enzymes in animals and plants, respectively. Most features of archaeal transcription — including the RNAP, general transcription factors that govern its activities and the DNA sequence elements with which they interact — are closely related to the eukaryotic RNAPII system. The archaeal transcription apparatus is likely to resemble the ancestral version of eukaryotic RNAPII and thus worthy of our attention not only because it is interesting in its own right, but also because it serves as highly tractable and thus extremely valuable model system.

Archaea are prokaryotic organisms that occupy a key position in the tree of life. The development of culture-independent sequencing techniques highlighted the abundance of archaea in diverse environments such as soils, deep-sea sediments and hydrothermal systems. Archaea are also well-recognized components of the human microbiome and provide a broader view on biodiversity. To date, archaea comprise at least four major superphyla, each of which comprises various phyla: Euryarchaeota (subdivided into group I and II), DPANN (Diapherotrites, Parv-, Aenigm-, Nano-, Nanohaloarchaeota, and others), TACK (Thaum-, Aig-, Cren-, Kor- and Bathyarchaeota) and ASGARD (Loki-, Odin-, Thor- and Heimdallarchaeota) [1–5]. Genetically very diverse, archaea use a single type of RNAP to transcribe all genes. However, lineage-specific RNAP subunits, such as Rpo8 and Rpo13, shed light on the acquisition of transcription function during evolution.

## Architecture and function of the archaeal RNAP subunits

**Figure 1.**
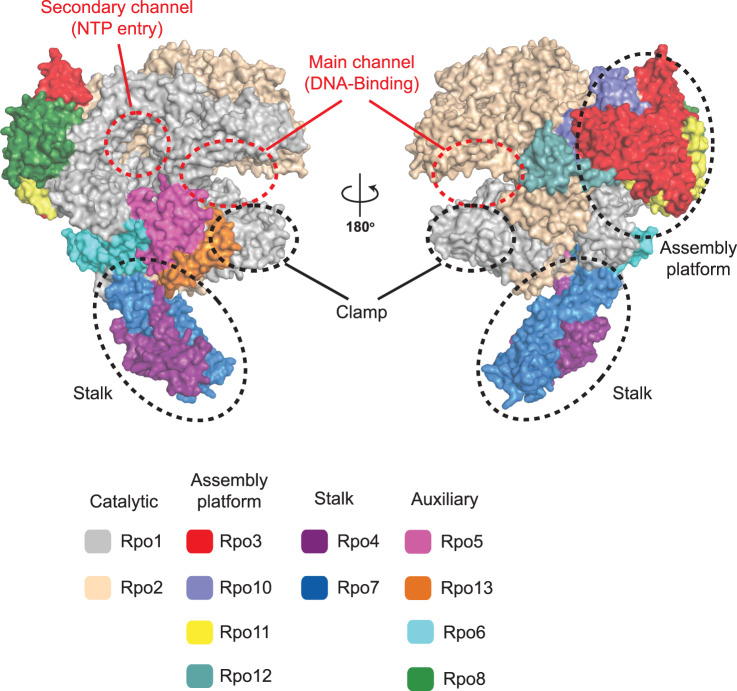
Structure of the archaeal RNAP. Overall architecture of the archaeal RNAP (subunits are colour-coded according to the key). The DPBB-1 and -2 comprising the catalytic centre reside in the two largest subunits Rpo1 and -2, respectively. Important structural features and motifs, including the RNAP assembly platform, stalk, clamp and the (main) DNA-binding channel and (secondary) NTP entry channel, are highlighted with dashed circles.

All cellular RNAPs share a subunit core whose ancestry predates the last universal common ancestor and thus the diversification into the lineages that have evolved into extant bacteria, archaea and eukaryotes [6]. The RNAP core is formed by five universally conserved subunits (Rpo1, 2, 3, 6 and 11 in archaea) and contains, in principle, all critical elements for transcription. In addition, RNAP subunits not conserved in bacteria play important roles for the assembly and stability of RNAP (Rpo10 and 12), its interactions with downstream DNA (Rpo5 and 13), the RNA transcript as well as the initiation factor TFE (Rpo4 and 7). RNAP subunits and their functions are summarized in Table 1 and Figure 1. The catalytic centre enabling phosphodiester bond formation and cleavage in all multisubunit RNAPs is formed between two structural motifs called double-psi β-barrels (DPBBs) residing in the large subunits (Rpo1 and Rpo2) [7,8]. In many archaea, the genes encoding the large RNAP subunits Rpo1 and Rpo2 are split into two open reading frames [8,9]. The two DPBBs acquired different functions crucial for the activity of extant RNAPs: One DPBB provides three carboxylate residues (aspartic acid) for the active site that chelate one of the two catalytic magnesium ions (Mg-A), while the second DPBB contributes two universally conserved lysine residues that facilitate interactions with nucleic acid and NTP substrates [10,11]. The overall RNAP core resembles a crab claw with a DNA-binding channel (aka main channel) between its pincers that leads the DNA template strand towards the active site (Figure 1). The NTP entry channel (aka secondary channel) connects the external milieu with the RNAP active centre allowing NTP substrates to enter the RNAP active site [12] and the RNA 3′-terminus to be extruded through it in backtracked transcription elongation complexes (ECs) (see below).

**Table 1:** Evolutionary conservation of RNAP subunits and general transcription factors Table summarizes the archaeal RNAP subunits, transcription initiation- and elongation factors, and indicates the homologous components in bacteria and eukaryotes. The column on the right indicates the molecular functions discussed in detail in the text. Note that the bacterial sigma-70 factor is functionally analogous to the TBP/TFB duo, while only sharing a very limited sequence similarity with TFB. Bacterial Gre factors are functionally analogous, but not homologous, to TFS/TFIIS transcript cleavage factors.

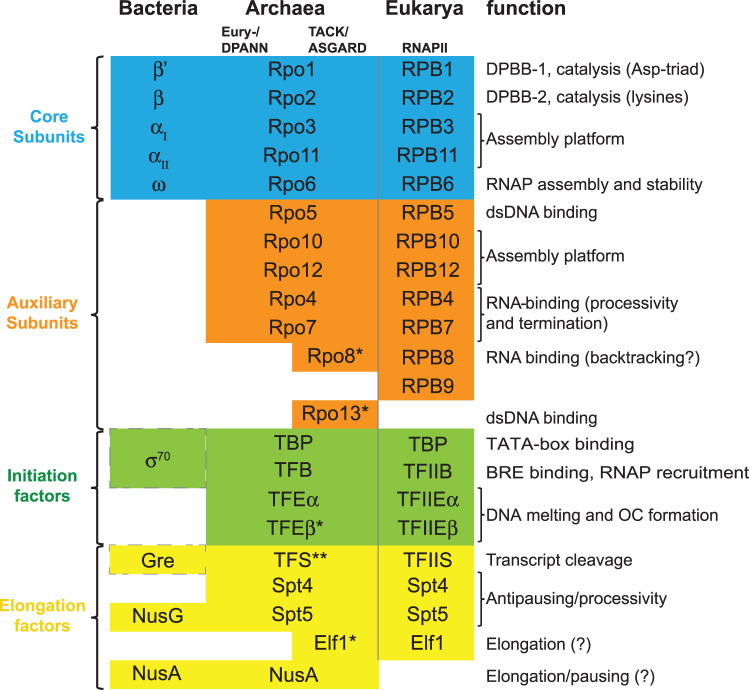

* Only found in some species.

** Archaeal TFS is evolutionarily related to RNAPII subunit RPB9 and to the transcript cleavage factor TFIIS in eukaryotes.

**Figure 2.**
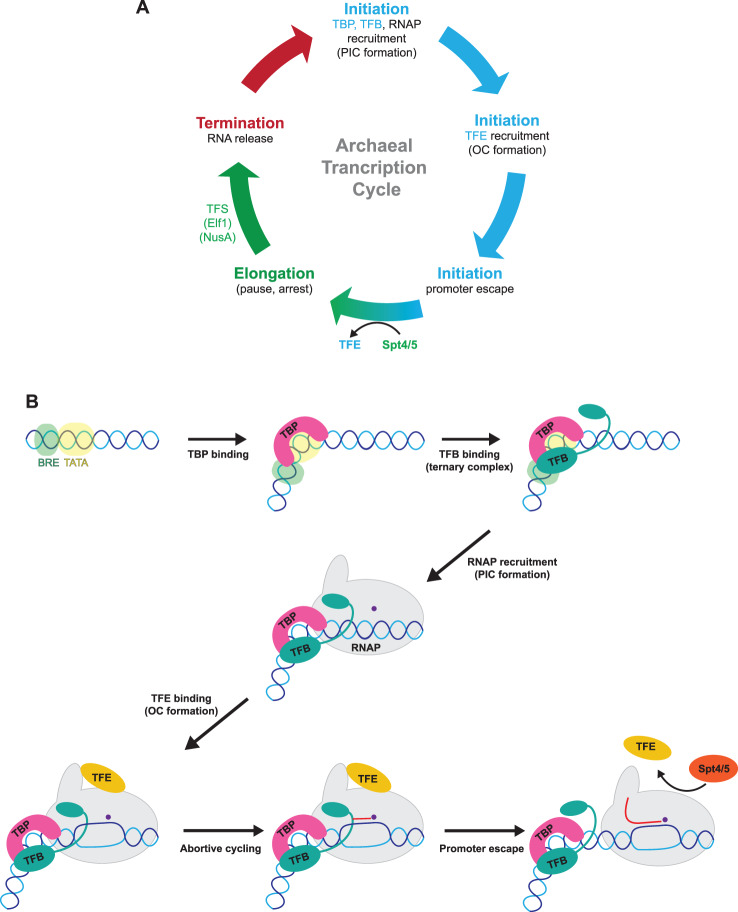
The archaeal transcription cycle. (**A**) The archaeal transcription cycle consists of initiation, elongation and termination phases during which RNAP is assisted by general transcription factors. (**B**) Transcription cascade. TBP (pink) and TFB (green) bind to the TATA-box and BRE promoter elements, respectively, forming a ternary complex. RNAP (grey) is subsequently recruited to form the minimal PIC. TFE (yellow) is recruited to the PIC, and enhances the transition between the CC and OC which occur concomitantly with DNA strand separation and formation of the transcription bubble. In the presence of NTP substrates, RNAP undergoes abortive initiation that produces 3–9 nt RNA species — also called abortive transcripts or nano-RNAs. The elongation factor Spt4/5 (orange) displaces TFE in a process coined factor swapping during promoter escape or early elongation. Template and non-template DNA strands are shown in dark blue and light blue, respectively. Catalytic Mg-A is shown in purple.

Like all molecular machines, the RNAP comprises a combination of rigid and flexible parts; the most prominent conformationally flexible domain of RNAP is the clamp. Movements of the clamp are conserved in all DPBB RNAPs and not only alter the width of the DNA-binding channel but also translate into the microenvironment of the active centre. During transcription, RNAP progresses through three distinct phases of the transcription cycle starting with initiation of transcription, elongation and termination with concomitant release of the transcript (Figure 2A). Interactions with the DNA template and general initiation- and elongation factors modulate the position of the clamp, resulting in distinct clamp closure states that reflect functional states of RNAP at different phases of the transcription cycle. In brief, FRET measurements on *Methanocaldococcus jannaschii* RNAP revealed that clamp opening is important (i) during transcription initiation for DNA melting and template strand loading into the active site enhanced by the initiation factor TFE, (ii) keeping the clamp closed in conjunction with the factor Spt4/5 during elongation enables high processivity [13,14]. In addition, an opening of the clamp accompanies pausing and is a prerequisite for bacterial transcription termination [15].

Rpo13 is the only archaea-specific RNAP subunit, and it is only conserved in species belonging to the Sulfolobales family of Crenarchaeota. Rpo13 is located at the downstream end of the DNA-binding channel and has been speculated to interact with the DNA template. The most prominent difference between bacterial and archaeo-eukaryotic RNAPs is the stalk domain comprising Rpo4 and Rpo7. The stalk interacts with the initiation factor TFE during initiation, and with the nascent RNA transcript during elongation via an oligonucleotide/oligosaccharide binding (OB) S1 domain residing in Rpo7. The interactions between the RNA and Rpo7 have been reported *in vitro*, they increase the processivity during elongation, and enable efficient termination at weak intrinsic terminator signals [16]. Rpo8, like Rpo7, contains an OB-fold [17]. The functional implications of this OB-fold are unclear; however, the location adjacent to the secondary channel suggests that it could interact with the 3′ segments of the RNA that are extruded through the pore in backtracked ECs [11,18]. Rpo8 is the only archeo-eukaryotic acquisition in the RNAP subunit repertoire that is not conserved in all archaea. While Rpo8 is present in species belonging to the TACK (Cren- and Korarchaeota) and ASGARD (Odin- and Heimdallarchaeota) superphyla, it is not conserved in euryarchaeal and DPANN species (Table 1) [19,20]. The eukaryotic homologue of the archaeal transcript cleavage factor TFS corresponds to the RNAPII subunit RPB9 as well as to the transcription factor TFIIS, a special case that is discussed in depth in section ‘RNAP backtracking, arrest and reactivation’. Overall, archaeal RNAPs and in particular the RNAPs of the TACK and ASGARD superphyla are closely related to eukaryotic RNAPII in terms of subunit composition.

## Factors and mechanisms enabling transcription initiation in archaea

While bacterial RNAPs require sigma factors to initiate transcription, archaeal RNAP utilizes the three general transcription initiation factors TBP (TATA-binding protein), TFB (Transcription Factor B) and TFE (Transcription Factor E) that are homologous to eukaryotic TBP, TFIIB and TFIIE [21–25], respectively. On the nucleic acid level, three consensus promoter elements direct the assembly of transcription initiation complexes on the promoter: the TATA-box (7–8 bp in length), B-recognition- (BRE, 3–5 bp in length) and the initiator (Inr) elements. The consensus DNA sequences of these elements is differentially conserved in the archaeal lineages; the TATA element is highly conserved, the BRE consensus is juxtaposed to TATA elements and enriched in A-residues, and the Inr (T(A/G)) is conserved in some archaea (e.g. *M. jannaschii*, *Methanolobus psychrophilus*, *Sulfolobus solfataricus* and *Haloferax volcanii*) but not in others (e.g. *Methanosarcina mazei*, *Thermococcus kodakarensis*) [26]. TATA and BRE serve to recruit transcription initiation factors. The sequence and role of the Inr is difficult to decipher because this motif overlaps extensively with start codon positions in many archaeal species such as *S. solfataricus* where most mRNAs are leaderless. A sequence preference for purines at the TSS preceded by a pyridine is a universal feature not only of DPBB RNAPs, but also of other RNAPs as it helps positioning the initiating NTP substrate [27]. A genome-wide comparison of transcript 5′-ends and the Inr motifs of the corresponding promoters revealed that the Inr is important for the exact positioning of the transcription start site TSS in *M. jannaschii* [28].

Archaeal TBP corresponds to the eukaryotic TBP core domain that binds to and distorts the TATA-containing promoter DNA by ∼90° (Figure 2B) [29,30]. TBP has an internal symmetry consisting of two repeats that are derived from an ancestral DNA-binding domain present in RNaseHIII [31,32]. The kinetics and stability of the TBP–DNA interaction differs significantly between archaeal species suggesting lineage-specific adaptation. For many archaea, the formation of a stable TBP:DNA complex requires TFB recruitment concomitant with TBP binding [29,33]. TFB, like its eukaryotic counterparts, consists of an N-terminal ZR (Zn-ribbon) domain connected by a flexible linker region to two cyclin fold domains at the C-terminus. The linker region itself comprises the B-reader and the B-linker motifs [34]. The orientation of the ternary TBP–TFB–DNA complex determines the directionality of transcription and relies on interactions between the second cyclin fold of TFB and the BRE upstream of the TATA-box [35]. The ZR domain of TFB interacts with the RNAP dock domain and recruits RNAP to the promoter forming a minimal DNA–TBP–TFB–RNAP pre-initiation complex (PIC). The B-linker penetrates deep into the RNAP and stabilizes the template DNA strand (TS) in the active site [34,36,37]. Many archaea, most prominently haloarchaea, utilize combinations of multiple TBP and TFB homologues, allowing different combinations of TBP–TFB which enable a certain degree of promoter specificity [38–40]. Additional TFB paralogues do not necessarily function the same way as canonical TFBs. TFB3, a TFB paralogue in *Sulfolobus* that is induced by UV-radiation and DNA damage, cannot replace the canonical TFB homologue, but rather appears to activate transcription in conjunction with canonical TFB in trans via a mechanism that is still poorly understood [41]. Recent insights into the genes under direct control of TFB3 provide now a basis for functional studies into the molecular mechanism of transcription activation by TFB3 [42,43].

To load the DNA TS into the RNAP active centre, the DNA strands are locally melted in a region 12 bp upstream of the TSS. This process is accompanied by large-scale conformational changes of the PIC that are referred as closed (CC) to open complex (OC) transition [21,44–46]. The initially melted region (IMR) shows an increased AT-content that might aid DNA melting in some, but not all archaea. DNA melting and OC formation are facilitated by the third archaeal initiation factor termed TFE. Canonical archaeal TFE and its eukaryotic counterpart TFIIE are composed of two subunits (α and β) [21]. TFEα and TFIIEα share the bipartite WH (winged helix) and ZR domain organization that interact with RNAP in a bidentate fashion: the TFEα ZR domain interacts with the RNAP clamp and stalk, whereas the TFEα WH domain interacts with the RNAP clamp coiled-coil (clamp-CC) domain [47]. The interactions of TFEα with both the stalk and clamp domains of RNAP together with interactions of TFE with the non-template strand (NTS) of the promoter DNA retain the clamp in the open conformation, and stabilize the transcription bubble, respectively, facilitating OC formation [45,47,48] (Figure 2B). In the presence of NTP substrates, the RNAP enters into abortive cycles of synthesis which release short RNA transcripts (2–15 nt) prior to the full extension of the RNA–DNA hybrid and escape from the promoter [46,49,50].

## Is TFEβ a global regulator of transcription?

The TFEβ subunit has a patchy phylogenetic distribution and is present in most group I euryarchaeota (with the exception of Thermoplasmata that lack both α- and β-subunits), TACK (missing in Korarchaeota) and ASGARD superphyla, but is absent from species from group II Euryarchaeota and DPANN [26,51]. TFEβ consists of an N-terminal WH- and a C-terminal cubane [4Fe–4S] cluster domain. The [4Fe–4S] cluster easily undergoes oxidative damage rendering TFE sensitive to oxidative stress. TFEβ expression levels vary dramatically with growth conditions and environmental stresses in *S. solfataricus*, unlike the remaining general transcription factors. Since TFE is a general factor and its activation of transcription varies considerably between promoters, the depletion of TFEβ has the potential to alter the RNA synthesis globally in *S. solfataricus*. In essence, modulation of OC formation provides an opportunity for the regulation of transcription, a mechanism which has previously been shown to operate in bacterial and eukaryotic transcription systems [26]. Interestingly, *H. volcanii* TFEβ and indeed all haloarchaeal homologues lack the [4Fe–4S] cluster that is essential for *S. solfataricus* TFEβ function. Nevertheless, in line with TFEβ being a bona fide general transcription factor, the deletion of the *H. volcanii* TFEβ results in the misregulation of approximately one-third of all transcription units [52]. The group II Euryarchaeota lacks TFEβ altogether and monomeric TFEα can fully support OC formation [23,36,48]. This broad, though patchy phylogenetic, distribution suggests that both TFEα and β subunits were present in the last archaea common ancestor (LACA) [26,52].

## Promoter escape: early transcription elongation

All DPBB RNAPs face similar mechanical engineering problems when entering the early elongation phase of the transcription cycle. A network of high affinity interactions between DNA-bound initiation factors (TBP, TFB and TFE) and RNAP are important to enable efficient recruitment to the promoter. However, these interactions need to be disrupted for RNAP to escape the promoter and enter processive transcription elongation. Structures of the initially transcribing complex of yeast RNAPII as well as recent cross-linking studies in *Pyrococcus* have shown that once the nascent RNA exceeds 5 nt in length, it collides with the TFB B-reader and B-linker domains, disrupting the interaction with and displacing TFB from the active site of RNAP [34,53,54]. Promoter escape of archaeal RNAP has not been well studied thus far and probably differs from its eukaryotic counterpart RNAPII with its drastically increased repertoire of initiation factors. Exonuclease and permanganate foot-printing studies revealed that promoter escape is initiated once the nascent RNA reaches 10 nt in length [46]. Once the elongating RNAP has reached register +15, the interactions between TFB and the DNA downstream of the TATA-box are disrupted [54]. An additional feature of the promoter escape is the swapping of initiation (TFE) and elongation factors (Spt4/5), both of which bind to overlapping binding sites on the RNAP clamp-CC motif in a mutually exclusive manner. This mechanism was initially discovered using biochemical and biophysical interaction analysis and transcription assays *in vitro* [47] and it is supported with the early recruitment of Spt4/5 to the vast majority of transcription units *in vivo* determined using chromatin immunoprecipitation (ChIP-seq) [28]. The association of Spt4/5 possibly induces allosteric changes in RNAP from an initiation- to elongation competent conformation. In line with this idea, single molecule FRET experiments showed that TFE and Spt4/5 exert opposing effects on the position of the RNAP clamp [14]. The global occupancy analysis revealed that a subset of non-coding RNA transcription units, including the ribosomal RNA operons and CRISPR (Clustered Regularly Interspaced Short Palindromic Repeats) loci, displayed a delayed Spt4/5 recruitment to the promoter, suggestive of an alternative promoter escape mechanism possibly reliant on additional uncharacterized transcription factors [28].

## Factors and mechanisms that enable efficient transcription processivity

A subset of evolutionarily conserved regulatory factors assist RNAP during transcription elongation by modulating the elongation rate and/or by improving the processivity (defined as polymerized nucleotides per initiation). Elongation factors belonging to the Spt4/5 family (the bacterial homologue of Spt5 is called NusG) stimulate transcription by binding to the RNAP clamp-CC on one side of the DNA-binding channel and to the RNAP gate loop on the other [13,55–58]. This locks the clamp into the closed state and seals the DNA-binding channel of RNAP, which counteracts the dissociation of the EC [13,59,60]. In addition, the interaction between the Spt5/NusG NGN (NusG N-terminal) domain and the template DNA enhances the annealing of the TS and NTS at the upstream edge of the transcription bubble and thereby suppress backtracking and pausing, which overall improves processivity and increases the elongation rate [61]. Bacterial NusG is monomeric, while in archaea and eukaryotes, NusG homologue Spt5 forms a heterodimer with a small ZR-containing protein Spt4. Spt4 not only exerts a stabilizing effect on the Spt5 NGN domain but also may functionally interact with the upstream DNA of the EC [13,59]. In addition to the NGN domain, Spt5 contains one (in archaea and bacteria) or several (in eukaryotes) KOW (Kyrpides–Ouzounis–Woese) domains. In bacteria and probably in archaea, the KOW domain interacts with the ribosomal protein S10 thereby physically coupling RNAP and the first co-translating ribosome, coordinating transcription and translation [55,62].

**Figure 3.**
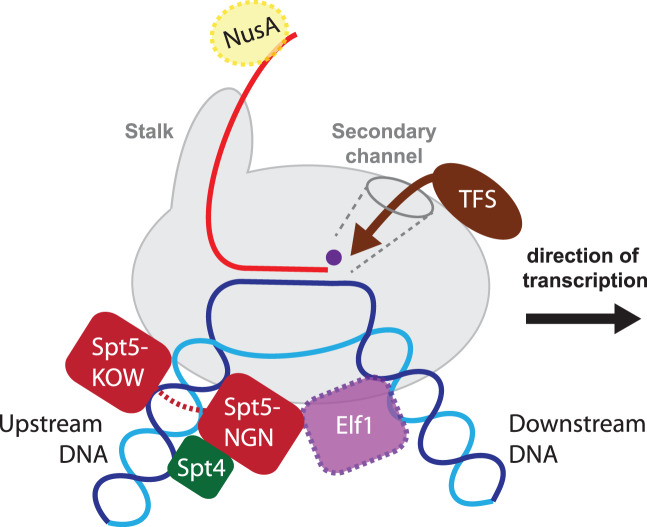
The archaeal transcription elongation complex. Schematic representation of the complete archaeal transcription elongation complex encompassing RNAP-DNA/RNA, TFS, Spt4/5 and possibly Elf1. The function of the cleavage and processivity factors are discussed in detail in the main text. The RNA-bound NusA is indicated beyond the tip of the RNAP stalk. Factors with unknown function is archaea are highlighted in dashed lines.

Structural insight into complete yeast transcription ECs encompassing RNAP, DNA, TFIIS, Spt4/5 and Elf1 reveals a striking reoccurring theme, by which the latter two elongation factors form entry and exit tunnels for the DNA and RNA strands [58,59]. Elf1 (Elongation factor 1) is a transcription elongation factor conserved in eukaryotes and several archaeal species [63]. Homologues of Elf1 have been identified in of the TACK (Cren-, Kor-, Aig- and Bathyarchaeota) as well as in the ASGARD superphylum (Table 1) [3,63,64]. Elf1 comprises a positively charged N-terminal α-helical tail, a structurally discrete ZR domain and a negatively charged unstructured C-terminal tail [59,65]. ChIP-Seq analyses in yeast demonstrate that Elf1 accompanies elongating RNAPII in similar manner to Spt4/5 [66], and *in vitro* transcription assays showed that Elf1 inhibits transcription elongation, possibly by interacting with downstream DNA via its N-terminal tail [59]. Elf1 is likely also part of the archaeal EC (Figure 3); however, the mechanism and function of Elf1 during transcription elongation in archaea remains enigmatic.

## A likeness between the Rpo4/7 RNAP stalk and NusA

**Figure 4.**
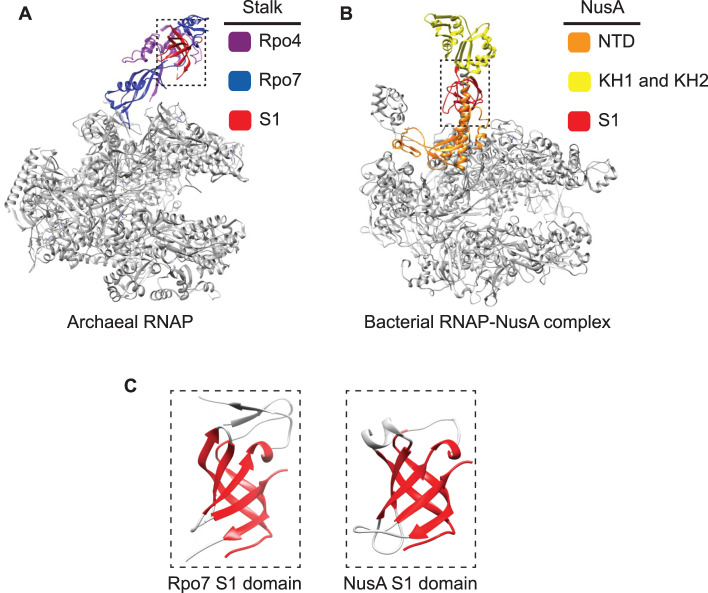
Structure comparisons of archaeal RNAP and the bacterial RNAP–NusA complex. (**A**) The archaeo-eukaryotic Rpo4/7 subunits form a stalk-like protrusion highly reminiscent of (**B**) the RNAP-bound bacterial elongation and antitermination factor NusA. The S1 domains of Rpo7 and NusA are highlighted in dashed lines. The insertion domains SI1, SI2 and SI3 of *E. coli* RNAP and regulatory C-terminal domain of NusA were omitted for clarity. (**C**) RNA-binding S1 domains of archaeal Rpo7 (PDB: 1GO3) and bacterial NusA (PDB: 1K0R).

The origin of the RNAP stalk — a hallmark feature of archaeal and eukaryotic RNAPs — is opaque, but a combination of recent structural and functional studies has revealed a striking resemblance to a bacterial elongation factor. The bacterial NusA (N-utilization substance A) interacts with the RNAP via the NusA N-terminal domain (NTD), and with RNA in at least two distinct ways that have different effects on transcription elongation. Interactions of the NusA S1 domain with an RNA hairpin enhances transcription pausing. In contrast, interactions between the NusA KH1 and -2 domains and the RNA nut (N-utilization target) sequence promote antitermination on ribosomal RNA operons [67]. This increases the elongation rate and renders the RNAP inert to the action of the termination factor rho [68]. All archaea encode one or several genes homologous to NusA, but archaeal NusA variants only encompass the two RNA-binding KH domains and not the N-terminal RNAP-interaction- and S1 domains altogether [69,70]. The archaeal RNAP stalk subunit Rpo7 includes an RNAP interaction domain, as well as an S1 domain that interacts with the nascent RNA transcript, which in turn modulates both transcription elongation and termination properties of the elongation complex [16] (Figure 4A). In combination, the RNAP interaction- and S1 domains of Rpo7 in conjunction with the two KH domains of archaeal NusA provide the complete domain complement of bacterial NusA [71]. Moreover, a recent structure of the bacterial RNAP–NusA complex shows that NusA forms an elongated stalk protruding from the RNAP proximal to the RNA exit channel, somewhat reminiscent of the archaeal and eukaryotic RNAP structures (Figure 4B) [72]. The possibility of a relationship between Rpo7 and NusA is enticing, and the S1 domains of archaeal Rpo7 and eukaryotic RPB7 and bacterial NusA are homologous [71] (Figure 4C). If indeed Rpo7 is homologous to NusA, an important question remains how the division of one polypeptide (NusA) in bacteria into two distinct (Rpo7 and NusA) polypeptides in archaea-altered NusA function.

## Transcription bubble maintenance by flexible RNAP motifs

**Figure 5.**
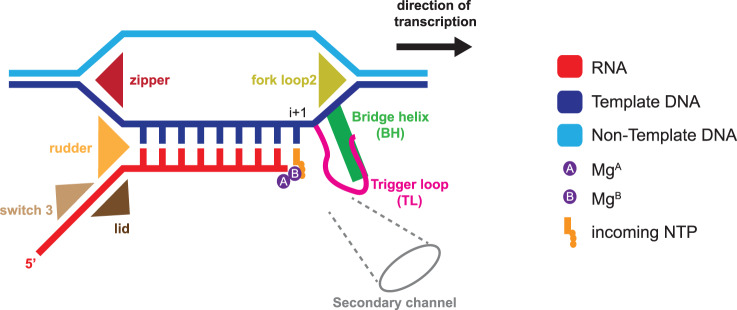
Flexible motifs enable the nucleotide translocation cycle of DPBB–RNAP. Schematic representation of RNAP active centre in the transcribing RNAP elongation complex (EC), the NTP insertion site corresponds to register i + 1. The motifs critical for RNA–DNA hybrid maintenance are shown as coloured triangles, and the trigger loop (TL) and bridge helix (BH) are shown in magenta and green, respectively, RNA, DNA strands, catalytic Magnesium ions and NTP substrate are coded according to the key.

During transcription elongation, RNAP translocates along the template DNA via a thermal Brownian ratchet mechanism [73–75]. The active centre of RNAP contains several polypeptide loops that were shown to be critical for the proper arrangement of the RNA–DNA hybrid and its stability during RNAP elongation. The downstream DNA stacks on the fork loop 2, which plays a critical role in double-strand DNA separation [76] (Figure 5). The lid serves as a wedge to facilitate RNA displacement by sterically blocking the formation of an overextended hybrid [76–78], while the rudder interacts with the RNA and overall stabilizes the EC [79]. Switch 3 binds to each RNA base in a nascent transcript as it dissociates from the RNA–DNA hybrid, stabilizing the EC [80,81]. Finally, the double-stranded DNA is reformed at the upstream edge of the transcript bubble by the zipper motif [11,77].

## RNAP backtracking, arrest and reactivation

Transcription elongation is a discontinuous process during which the EC pauses relatively frequently. This pausing can be induced in DNA sequence-dependent fashion (e.g. poly-A stretches in the TS) or by roadblocks such as DNA-bound proteins (e.g. chromatin proteins) or DNA lesions [82–85]. Upon pausing, RNAP can move in a retrograde direction along the DNA, which is referred to as backtracking. During this process, the RNA–DNA hybrid maintains its 8–9 bp length, while one or more nucleotides of the RNA 3′-end are displaced from the downstream edge of the RNA–DNA hybrid out of the active site rendering the backtracked EC catalytically inert. If backtracking proceeds further, longer RNA 3′ segments are extruded from the RNAP through the secondary channel. Backtracked ECs pose a severe problem for the cell since they act as roadblocks for upstream transcription ECs and replication forks, which can lead to double-stranded DNA breaks compromising genome integrity [86]. Transcript cleavage factors resolve this conflict by inducing an endonucleolytic cleavage activity inherent in DPBB RNAPs. This generates a new RNA 3′-end conducive to RNA polymerization and transcription elongation can commence.

While archaea and eukaryotes utilize evolutionary related factors, TFS and TFIIS, respectively, the non-homologous bacterial Gre factors carry out the same function while providing a stunning case of convergent evolution [87] (Table 1). With the exception of the euryarchaeon *Methanopyrus kandleri*, TFS is conserved in all archaeal species [88]. Both TFS and TFIIS are evolutionarily related to RPB9-like subunits of eukaryotic RNAPs, but while RPB9 subunits are stably incorporated into RNAP, TFS/TFIIS associate with their cognate RNAP in a reversible fashion (Table 1) [89]. All transcript cleavage factors position two acidic residues at the tip of an elongated insertion domain through the secondary into the RNAP active site. The carboxylate moieties stabilize a magnesium ion required for the stable coordination of a water molecule that carries out a nucleophilic attack on the RNA phosphodiester bond triggering RNA cleavage [89–91].

## Functional diversification of archaeal transcript cleavage factors

**Figure 6.**
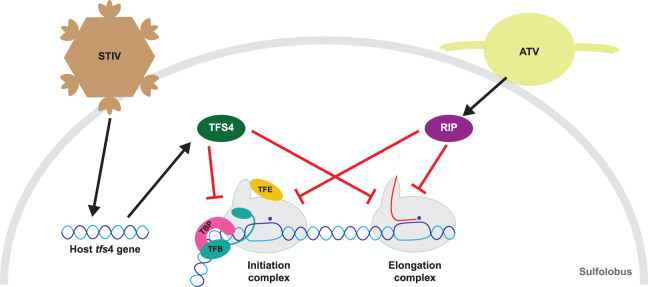
Global transcription inhibition in the virus–host relationship. Both host- (TFS4) and virus-encoded factors (RIP) can directly associate with the archaeal RNAP and efficiently shut down transcription on a genome-wide scale. The *S. solfataricus* transcript cleavage factor homologue TFS4 interacts with RNAP like other cleavage factors such as TFS1 through the NTP entry channel. Rather than promoting transcription elongation, TFS4 dramatically lowers the affinity of RNAP for NTP substrates thereby inhibiting catalysis, and induces allosteric changes that destabilize RNAP-nucleic acid interactions. TFS4 expression is repressed during normal cell growth but highly induced by infection with the STIV. In comparison, the ATV that infects Sulfolobales encodes the small protein RIP, which is derived from a viroid coat protein but has evolved into a potent inhibitor of the archaeal RNAP. RIP binds to the RNAP clamp in the DNA-binding channel, locks the clamp into a fixed position and inhibits RNAP activity in a global fashion. Both TFS4 and RIP inhibit transcription in a DNA sequence-independent fashion, i.e. they repress host as well as virus promoters. While the former has been speculated to provide a survival mechanism for the infected host akin to persistence, the latter probably serves the virus by preventing or attenuating the activation of cellular antiviral type III-B CRISPR–Cas system.

Several archaeal species encode more than one TFS paralogue, e.g. the genome of the crenarchaeon *S. solfataricus* includes four apparent TFS paralogues (TFS1 to 4). While TFS1 carries out the canonical transcript cleavage function, TFS4 has evolved into a highly potent RNAP inhibitor [91]. TFS4 shares a high degree of sequence similarity with TFS1 but lacks the catalytic acidic (DE) residues required for transcript cleavage activity. Rather, three lysine residues replace the acidic residues at the tip of the insertion domain. TFS4 binding to RNAP destabilizes transcription initiation and ECs which suggests that it exerts an allosteric effect that compromises the interactions between RNAP and the nucleic acid scaffold. These conformational changes are likely characteristics for all DPBB RNAP and related to the mechanism by which the bacterial regulator Gfh1 inhibits RNAP activity [92,93]. In addition to the allosteric mechanism, TFS4 acts as a competitive inhibitor for NTP binding to RNAP, possibly by sterical blockage as suggested by its binding site within the secondary channel. Expression of the *tfs4* gene is not detectable under standard growth conditions. However, infection with STIV (*Sulfolobus* turreted icosahedral virus) leads both to a dramatic increase in TFS4 expression, and induces a dormant state in the infected cell [94] (Figure 6). TFS4 is likely to play a key role in this process, since the ectopic overexpression of a TFS4 variant is sufficient to induce a severe growth retardation in line with its potent inhibitory effect on global transcription. Our understanding of the biological function of TFS4 during infection leaves much room for improvement, but it seems likely that the inhibition of transcription is an antiviral response that enables host survival by persistence. This is a survival strategy employed by bacteria in response to bacteriophage infection; the infected cells enter a hiatus to inhibit virus proliferation often in conjunction with additional, more active, defence mechanisms [95].

## Global inhibition of transcription in the host–virus arms race

TFS4 is a host encoded archaeal transcription factor that inhibits transcription on a global level in response to viral infection [91]. Surprisingly, archaeal viruses themselves use a very similar strategy to their own advantage. Archaeal cells are exposed to a plethora of viruses in their natural environment, and an ongoing arms race between the two has shaped the relationship between them [96]. One of the primary antiviral defence mechanisms of the archaeal hosts is provided by CRISPR–Cas systems [97]. Sophisticated viral counter measures involve the subjugation of the host transcription machineries, as well as strategies to stay ‘under the radar’ of surveillance mechanisms including the type IIIb CRISPR–Cas system that is triggered by active transcription [98–100]. Encounters between the *Acidianus* two-tailed virus (ATV) and *S. solfataricus* are witnessed by the presence of several ATV genome-derived CRISPR spacers in the hosts’ CRISPR arrays, including sequences mapping to a small gene called ORF145 [101]. ORF145, also called RIP (RNAP inhibitory protein), binds tightly to the inside of the DNA-binding channel of the host RNAP, thereby locking the RNAP clamp into a fixed position [102]. This counteracts the formation of transcription initiation complexes and inhibits abortive and productive transcription (Figure 6).

The interaction of RIP with RNAP differs in a fundamental way from TFS4, but the outcome is surprisingly similar. RNAP-nucleic acid complexes are destabilized and transcription initiation and elongation are inhibited. Because RIP, like TFS4, binds directly to RNAP, both host and virus promoters are inhibited in a global fashion. While the regulatory rationale behind this mechanism is still unclear, it is likely that the inhibition of transcription attenuates or even prevents the activation of the type IIIb CRISPR–Cas system and expression of anti-ATV CRISPR RNAs, while still enabling transcription on viral genes required for virus proliferation [102].

## Mechanisms and factors that enable transcription termination

**Figure 7.**
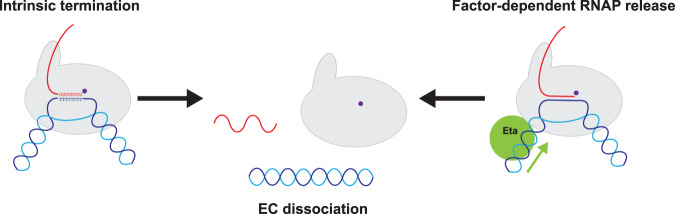
Transcription termination and RNA release. Schematic representations of transcription termination in archaea. Termination events that do not rely on exogenous factors are known as intrinsic termination. In archaea, intrinsic termination does not rely on secondary structures in the transcript such as RNA hairpins. Rather, a poly-U stretch seems sufficient to enable termination *in vitro* and *in vivo*. Recently, the first archaeal termination factor, Eta, has been shown to enhance RNA transcript release from stalled ECs. Eta is a Ski2-like DEAD box helicase that in an ATP-hydrolysis-dependent fashion translocates along the DNA; upon impact with the RNAP from the upstream direction the transcript is released, the TEC dissociates, and transcription is terminated.

Transcription termination not only defines the nascent 3′ terminus of the RNA transcript but is important to prevent transcription read through of RNAP from upstream into the adjacent transcription units in the densely crowded environment of small archaeal genomes. Despite its biological significance, transcription termination remains one of the least understood processes of gene expression in archaea. *In vitro* and *in vivo* studies have shown that euryarchaeal RNAPs are capable of terminating transcription directed by short poly-U stretches and unaided by exogenous factors, a property reminiscent of the eukaryotic RNAPIII system (Figure 7) [103–109].

An unbiased mapping of RNA 3′-ends in a euryarchaeal- (*M. mazei*) and crenarchaeal (*S. acidocaldarius*) species using Term-seq has provided an overview of RNA 3′-ends *in vivo* on a genome-wide scale [110]. However, Term-seq alone cannot discriminate between genuine transcription termination sites and RNA 3′ ends resulting from RNA processing; therefore, additional prior information, such as high-resolution RNAP occupancy profiling (ChIP-seq) and -transcriptome mapping (RNA-seq) and the position of the RNA 3′ end relative to operon structures and stop codons (at the end of ORFs), needs to be taken into account for a rigorous and meaningful analysis. Overall, the archaeal Term-seq study supports the notion that transcription terminates immediately downstream of uridine-rich sequences but also highlights additional, lineage-specific sequence features in *M. mazei* and *S. acidocaldarius* that are not accounted for in current models of transcription termination [110]. Termination in about one-third of genes in either system is enabled by multiple terminator elements, resulting in variations of non-coding 3′-untranslated regions (3′-UTRs) with differing lengths that could be involved in the regulation of gene expression by small non-coding RNAs. Alternatively, or in addition, transcription termination in archaea could be less precise compared with prototypical bacterial intrinsic terminators, possibly due to the lack of any RNA hairpin secondary structures in archaeal terminators.

Little is known about factor-dependent transcription termination mechanisms in archaea. In *T. kodakarensis*, the Ski2-like RNA helicase Eta (Euryarchaeal Termination Activity) is a DEAD box helicase that is recruited to stalled transcription complexes via interactions with the DNA immediately upstream of the arrested RNAP [111,112]. Eta translocates along the DNA in an ATP-dependent fashion, pushes the EC forward and thereby releases the nascent RNA (Figure 7). It is important to point out that Eta, unlike other transcription termination factors in bacteria and eukaryotes, is not essential for cell viability and does not trigger transcription termination of actively elongating RNAPs [112]. Eta's properties suggest that it is not a general transcription termination factor but rather likely to be a component of the DNA damage response akin to the Mfd factor in bacteria [113,114].

## Future perspectives

RNAPs are among the most well-studied molecular machines of life. The initiation phase of transcription has been characterized over the last two decades. These studies have elucidated the structure, function and detailed mechanisms that govern the archaeal PIC. Many studies have identified positive and negative transcription factors that enhance or prevent its recruitment of the PIC. While the structure and mechanisms of elongation factors like TFS and Spt4/5 are reasonably well understood *in vitro*, a thorough understanding of how these factors influence transcription *in vivo* just starts to emerge. An integrated, genome-wide view of transcription in archaea shows promise to bring to light more sophisticated mechanisms of transcription regulation beyond the initial recruitment, probably involving promoter escape and transcription processivity during the elongation phase of the transcription cycle.

Recent discoveries of virus and host encoded global inhibitors of RNAP transcription have shed light on novel molecular mechanisms and regulatory strategies that seemingly play a key role in the host–virus arms race. Finally, our field is coming to terms with the fact that the chromatin structure, histone-based or otherwise, plays an important role in gene regulation in archaea. Novel approaches, including high-throughput sequencing techniques, live cell imaging, as well atomic-resolution cryo-electron microscopy, will lead to key discoveries and a new dawn of archaeal gene expression, with an ever more detailed understanding of transcription from the molecular to the systems level.

## Summary

- The catalytic centre or the archaeal RNAP is formed between two DPBB.
- Combined ChIP-seq and RNA-seq analyses reveal the genome-wide organization of transcription and generate new mechanistic hypotheses that can be tested *in vitro*.
- The general transcription initiation factor TFEβ has the potential to regulate transcription globally in response to environmental stresses.
- Transcription elongation is modulated by RNAP subunits (Rpo4/7) and transcription factors (Spt4/5, TFS1 and likely Elf1).
- The RNAP stalk subunit Rpo7 shows an intriguing structural and functional similarity to the bacterial pausing/antitermination factor NusA.
- The expression of the transcript cleavage factor paralogue TFS4 is induced by STIV virus infection and acts as a powerful global inhibitor of RNAP in *S. solfataricus*.
- The ATV virus-encoded regulator RIP binds directly to RNAP and results in the global inhibition of transcription; thus both host- and virus-encoded RNAP-binding transcription factors globally inhibit or attenuate total RNA synthesis.
- Genome-wide mapping of transcript 3′-ends changes our view on the sequence context of archaeal transcription terminators.
- RNAPs stalled by DNA-damage can be efficiently removed by the termination-like factor Eta.

## Abbreviations

ATV: *Acidianus* two-tailed virus
CC: closed
CRISPR: Clustered Regularly Interspaced Short Palindromic Repeats
DPBB: double-psi β-barrel
ECs: elongation complexes
Elf1: Elongation factor 1
Eta: Euryarchaeal Termination Activity
IMR: initially melted region
Inr: initiator
KOW: Kyrpides–Ouzounis–Woese
NTD: N-terminal domain
NTS: non-template strand
NusA: N-utilization substance A
OB: oligonucleotide/oligosaccharide binding
OC: open complex
PIC: pre-initiation complex
RIP: RNAP inhibitory protein
RNAP: RNA polymerase
STIV: *Sulfolobus* turreted icosahedral virus
TBP: TATA-binding protein
TFB: transcription factor B
TFE: transcription factor E
WH: winged helix
